# Editorial: Applications of Herbal Medicine to Control Chronic Kidney Disease

**DOI:** 10.3389/fphar.2021.742407

**Published:** 2021-09-07

**Authors:** Jianping Chen, Karl W. K. Tsim, Ying-Yong Zhao

**Affiliations:** ^1^ Shenzhen Key Laboratory of Hospital Chinese Medicine Preparation, Shenzhen Traditional Chinese Medicine Hospital, The Fourth Clinical Medical College of Guangzhou University of Chinese Medicine, Shenzhen, China; ^2^ Division of Life Science and Center for Chinese Medicine, The Hong Kong University of Science and Technology, Hong Kong, SAR China; ^3^ Faculty of Life Science and Medicine, Northwest University, Shaanxi, China

**Keywords:** chronic kidney disease, herbal medicine, mechanism, active components, safety usage

## Introduction

Chronic kidney disease (CKD) is a leading cause of life lost worldwide (Chen et al., 2019). The contributing factors to progression of CKD include parenchymal cell loss, chronic inflammation, fibrosis and reduced regenerative capacity of kidney (Ruiz-Ortega et al., 2020). The complications of CKD, e.g. anemia, are associated with increased risks of death (Fishbane and Spinowitz, 2018). The prevalence and mortality of CKD are rapidly increasing, illustrating the shortcomings of current therapeutic approaches. Research aiming to identify novel therapies for CKD is required to prevent disease progression and to prevent death. In recent years, herbal medicine has demonstrated its potential as an alternative therapy to treat numerous diseases, and which is attracting greater attention and being applied to control CKD. However, some of herbal medicines have been found to be nephrotoxic when incorrectly utilized. Thus, the proper usage of herbal medicine is necessary to avoid the nephrotoxicity. The goal of this research topic is to provide a forum to advance research of herbal medicine towards CKD therapies. Thirty-seven contributions covering the listed research topics have been submitted to this special issue.

## Reviews of Herbal Medicine in Prevention of Kidney Disease

Zhao et al. summarized recent studies on the efficiency of Chinese herbal medicine commonly used for the treatment of kidney disease. As a conclusion, Chinese herbal medicine has been shown to have effects of anti-inflammatory, anti-oxidative, anti-apoptotic, autophagy, and anti-fibrotic: these pharmacological properties could account for the therapeutic approach in treating CKD. Given the critical role of mitochondrial dysfunction in developing CKD, Tian et al. have summarized the efficiency of herbal formulae on mitochondrial dysfunction, providing novel insights into the rationale in developing new drugs to prevent and/or to treat CKD. Supporting this notion, a mini review by Li et al. has summed up the therapeutic effect of herbal medicine in CKD via targeting mitochondrial dynamic. Anemia is one of the most common complications in CKD patients. Here, Li et al. have performed meta-analysis to assess the efficacy of a herbal formula, Jianpi Bushen, in treatment of patients suffering from CKD anemia. This study demonstrated that Jianpi Bushen showed good efficacy in treating CKD anemia. In parallel, Feng et al. have summarized clinical outcomes of herbal medicine in treating idiopathic membranous nephropathy, one of the main types of CKD. This article has led to a new idea for treatment of idiopathic membranous nephropathy. In addition, Yu et al. have performed a network meta-analysis to investigate the efficacy of Chinese herbal injections in treating primary nephrotic syndrome. This study supports the clinical usage of herbal injection in clinic. A review by Shao et al. has summarized the pharmacological effects of herbal medicines in progression of autosomal dominant polycystic kidney disease, which paves a direction to develop effective herbal drugs for therapeutic usage. In summary, these review articles are providing different lines of scientific evidence supporting efficacy and mechanistic action of herbal medicine in CKD and its associated diseases.

## Herb and Herbal Formula for CKD

Herbal medicine, either as a single herb or in a formulated mixture, has been reported to exhibit protective role in CKD. Kidney fibrosis is the common final pathway of CKD. In this topic, three articles have focused on therapeutic effects of herbal extracts against renal fibrosis in rats. Wang et al. have performed lipidomic analysis to reveal the effect of *Polyporus umbellatus* extract on adenine-induced lipid metabolism disorder in rats. Their findings suggest that the extract of *P. umbellatus* could protect renal fibrosis by regulating fatty acyl metabolism. Sari et al. have elucidated the efficacy of *Centella asiatica* extract in restoring kidney fibrosis in an unilateral ureteral obstruction model. The results showed that treatment with *C. asiatica* extract could improve kidney fibrosis by reducing mesenchymal transition, collagen deposition and inflammation. In addition, Ning et al. have found that the treatment with genistein, an isoflavone, was able to increase expression of renal alkylation repair homolog 5, as well as to reduce expression of RNA m6A levels in unilateral ureteral occlusion-induced renal fibrosis. Besides, He et al. have shown that the in-take of rhein and curcumin mixture, major active ingredients of Bu-Shen-Huo-Xue herbal formula, could markedly improve renal fibrosis in CKD rats.

Recently, gut microbiota have been proposed to contribute development and progression of CKD. In this special issue, four articles have presented the roles of herbal medicine in remodeling dysbiosis of CKD rats. Al-Asmakh et al. have explored potential effects of gum acacia, a natural gum consisting of hardened sap of two species of acacia tree, i.e. *Acacia senegal* and *Vachellia* (*Acacia*) *seyal*, on gut microbiome, particularly in the amount of short chain fatty acids, and which further illustrated its beneficial effects in CKD rat model. These results support that the renal protective function of gum acacia, and the mechanism of which may be involved in improving growth of beneficial bacteria in gut, and thereafter which stimulates release of short chain fatty acids. Tu et al. have demonstrated that the extract of *Abelmoschus manihot* could protect renal damage by remodeling gut dysbiosis and inhibiting microinflammation triggered from intestinal metabolites. These findings provide information on the role of *A. manihot* in delaying CKD progression. Moreover, Guan et al. have explored the renal protection of herbal mixture containing *Scutellaria baicalensis* and *Sophora japonica*. This herbal mixture was able to low blood pressure and to reverse renal injury in a rat model of hypertensive nephropathy. The involvement of microbiota, short chain fatty acids and metabolites in gut has been proposed to be responsible for action of the herbal mixture. Lastly, Li et al. have demonstrated the effect of Zhen Wu Tang, a classic herbal decoction from ancient China, in ameliorating kidney injury in rats having immunoglobulin A nephropathy. Again, the adjustment of intestinal flora and the restoration of metabolism homeostasis are proposed to be the outcome of Zhen Wu Tang treatment.

Moreover, three articles have focused on other aspects of therapeutic benefits in treating CKD. First, exosomal miRNA profiling has been revealed in CKD rats. Liu et al. have found that 4 exosomal miRNAs were markedly disturbed, and which could be modulated by Jian-Pi-Yi-Shen formula, a Chinese herbal decoction, in adenine-induced CKD rats. Second, the positive response of a herbal formula Yi-Qi-Jian-Pi-Xiao-Yu-Xie-Zhuo on muscle atrophy in CKD rats has been investigated, and the mechanism of which has been proposed to be involved in modulating IGF-1/PI3K/Akt signaling (Xia et al.). The article by Zhou et al. has shown that Jian-Pi-Yi-Shen herbal formula could restore renal oxidative injury by activating nuclear factor (erythroid-derived 2)-like 2 signaling in CKD rats.

## Herbal Medicine for Diabetic or Other Kidney Diseases

Diabetic kidney disease is one of the leading causes of end-stage renal disease, and it is therefore of great importance to delay the progression of diabetic kidney disease. Three articles are presented in this topic assessing renal protective effects of herbal medicine in diabetic animal models. Wang et al. have demonstrated that Bu-Shen-Huo-Xue herbal decoction effectively regulated high blood glucose and renal function in diabetic mice, and the underlying mechanism of which was proposed to have an involvement of inhibition of Rac1/PAK1/p38MAPK signaling. Another study by Li et al. have revealed that Tang Shen herbal formula could attenuate diabetic kidney injury and reduce pyroptosis of tubular epithelia via TXNIP-NLRP3-GSDMD axis in diabetic rats. The third article by Xuan et al. is revealing the effect of Yiqi Jiedu Huayu herbal decoction in diabetic nephropathy. Treatment with Yiqi Jiedu Huayu herbal decoction improved renal pathological damage and renal function, and the mechanism of which might be related to downregulation of mTOR pathway via PI3K/Akt and AMPK pathways. Apart from herbal decoction, Yang et al. have reported the effect of Huidouba, a Tibetan medicine derived from the nest of *Atypus karschi*, in diabetic nephropathy. Having an intake of Huidouba could down regulate the expression of Nox4 and relieve the oxidative injury in podocytes and proteinuria of diabetic nephropathy rats. Taken together, the aforementioned articles provide possible therapeutic strategies for treatment of diabetic kidney disease.

In addition, the therapeutic functions of herbal medicine on other kidney diseases have included here. Zhen-Wu-Tang, a well-known traditional Chinese herbal formula, restored renal dysfunction in immunoglobulin A-mediated nephropathy rat model, and the action of was revealed to be triggered by regulating exosome secretion in inhibiting NF-κB/NLRP3 pathway (Li et al.). Chao et al. have employed untargeted lipidomics to reveal action mechanism of *Orthosiphon stamineus* extract in treating nephrolithiasis. They found that the anti-stone effect of *O. stamineus* extract was closely related to glycerolphospholipid-mediated oxidative stress and inflammatory response. In parallel, Xiong et al. have investigated the effect of I-BET151, a small-molecule inhibitor targeting the bromodomain and extra-terminal (BET) protein, on the development of hyperuricemic nephropathy, which supports the notion that BET inhibition may have therapeutic potential in prevention and treatment of the problem.

## Active Ingredients in Herbal Medicine for CKD

Ten articles are focused on identifying active ingredients within herbal medicine to treat CKD. Fucoidan, a natural compound of *Laminaria japonica*, was reported to ameliorate renal injury-related calcium-phosphorous metabolic disorder and bone abnormality in CKD mineral and bone disorder rats by targeting FGF23-Klotho signaling axis (Liu et al.). These data provide pharmacological evidence of fucoidan in the treatment of this disease. Ning et al. have shown that genistein could restore renal fibrosis by increasing alkylation repair homolog 5 to regulate epithelial-to-mesenchymal transition in unilateral ureteral occlusion-induced animal model. The result provides new insight into the function of m6A modification in CKD progression. Emodin, the most important component of *Rheum palmatum*, was being prepared in nanoparticles to form an emodin-nanoparticle system. This combination possessed higher stability and better colon adhesion in therapeutic effect on CKD (Lu et al.). He et al. have revealed that rhein and curcumin had a synergistic effect in ameliorating CKD, which provides an important explanation on the synergy of rhein and curcumin from a pharmacokinetic viewpoint. Tu et al. have demonstrated that total flavones, containing rutin, hyperoside, isoquercitrin, and quercetin extracted from *A. manihot*, could regulate the amounts of gut microbiota and its metabolites, and which inhibit microinflammation by targeting autophagy-mediated macrophage polarization in CKD rats. Saikosaponin D, a triterpene saponin isolated from *Bupleurum falactum*, was shown to inhibit peritoneal fibrosis in rats by regulating TGFβ1/BMP7/Gremlin1/Smad pathway (Liu et al.). Astragaloside II protected podocyte injury and mitochondrial dysfunction in diabetic rats, possibly *via* a co-modulation of nuclear factor (erythroid-derived 2)-like 2 and PTEN-induced putative kinase signaling (Su et al.). Li et al. have shown that scutellarin, a biologically active flavonoid derived from *Erigeron breviscapus*, restored renal injury *via* increasing cellular communication network factor 1 expression and suppressing nucleotide-binding oligomerization domain-like receptor protein 3 inflammasome in hyperuricemic nephropathy mice. Cui et al. have investigated the effect of oleuropein, an active ingredient of *Ilex pubescens*, on acute kidney injury model. Their findings showed that oleuropein attenuated lipopolysaccharide-induced acute kidney injury both *in vitro* and *in vivo* by suppressing dimerization of toll-like receptor. Zhou et al. have reported that glycyrrhetinic acid, a bioactive component of *Glycyrrhiza uralensis*, protected renal tubular cells against oxidative insult. The regulation of JNK-connexin 43-thioredoxin signaling is proposed to play a crucial role in this action. Taken together, these findings can offer promising candidates for new drugs against CKD.

## Clinical Studies

Two clinical studies here in this issue have highlighted application of herbal medicine on kidney disease. The first study by Mao et al. was a multicenter, double-blind, double-dummy, randomized controlled trial aiming to assess efficacy and safety of Bupi Yishen Chinese herbal formula in patients with non-diabetic CKD at stage 4. The results indicated that the herbal formula possessed protective effects on non-diabetic patients with CKD in first 12 weeks and over 48 weeks. Another study by Guo et al. have analyzed a retrospective population-based cohort to evaluate the long-term effect of using Chinese herbal medicine among incident pre-dialysis patients with diabetic kidney disease. The findings suggest that using Chinese medicine among pre-dialysis diabetic nephropathy patients is seemed to be feasible. Based on these two studies, a well-designed clinical study is still needed to evaluate the beneficial effect of herbal medicine in clinics.

## Nephrotoxicity of Herbal Medicine and Its Prevention

Three articles in this topic explored the nephrotoxicity of herbal medicine and its prevention. First, Xu et al. have summarized the mechanism underlying nephrotoxicity of compounds from herbal medicine, which may help in discovering the biomarkers of renal injury and developing preventive strategies. Aristolactam I, an active component from herbs, is considered to be the most important constituent causing aritolochic acid nephropathy (Bastek et al., 2019). Deng et al. have shown that mitochondrial iron overload-mediated antioxidant system could inhibit the aristolactam-induced ferroptosis in HK-2 cells. Targeting Nrf2-HO-1/GPX4 antioxidative system can be an important intervention strategy to prevent aristolactam-induced nephropathy. Besides, Gao et al. have shown the protective effects of astragaloside IV, a major saponin from *Astragalus* root, on chronic nephrotoxicity. The results showed that astragaloside IV alleviated tacrolimun-induced chronic nephrotoxicity by enhancing p62 phosphorylation, thereby facilitating Nrf2 nuclear translation, and then decreasing ROS accumulation and renal fibrosis. Overall, these studies provide possible strategies to reduce renal toxicity and to ensure the safety of clinical medication; however, which has to be confirmed by further research.

## Conclusion

This research topic collects 37 articles including a wide range of studies in the field of herbal medicine to control CKD. These studies improve our understanding on the mechanisms of herbal medicine in treating CKD and its related diseases. Potential therapeutic targets are also proposed for the prevention and treatment of CKD. Moreover, several active ingredients in herbal medicine are identified, which provides potential candidates for future drug development. The nephrotoxicity of herbal medicine and its prevention are also highlighted here. Besides, clinical studies support beneficial role of herbal medicine in improving CKD. Thus, this topic can provide effective evidence to promote the development of herbal medicine as therapeutic use to treat CKD, and such that which may help to prolong the survival of patients and reduce the burden of CKD.
